# Community and Hospital‐Based Laboratory Surveillance for Influenza, Respiratory Syncytial Virus, and SARS‐CoV‐2 During the 2023–2024 Season, Lyon, France

**DOI:** 10.1002/jmv.70549

**Published:** 2025-08-29

**Authors:** Cécile Chauvel, Jean‐Sébastien Casalegno, Benoit Visseaux, Vincent Vieillefond, Stéphanie Haim‐Boukozba, Vincent Enouf, Emmanuel Chanard, Margaux Fabre, Marie‐Anne Rameix‐Welti, Antoine Oblette, Jean‐Marc Giannoli, Juliette Paireau, Laurence Josset, Bruno Lina, Alexandre Gaymard, Simon Cauchemez, Florence Morfin, Marta C. Nunes, Antonin Bal

**Affiliations:** ^1^ Center of Excellence in Respiratory Pathogens (CERP) Hospices Civils de Lyon (HCL) Lyon France; ^2^ Centre International de Recherche en Infectiologie (CIRI), Équipe Santé Publique, Épidémiologie et Écologie Évolutive des Maladies Infectieuses (PHE3ID), INSERM U1111, CNRS UMR5308, ENS de Lyon Université Claude Bernard Lyon 1 (UCBL) Lyon France; ^3^ Laboratoire de Virologie, Centre National de Référence des Virus des Infections Respiratoires, Institut des Agents Infectieux Hospices Civils de Lyon Lyon France; ^4^ Laboratoire VirPath, Centre International de Recherche en Infectiologie (CIRI), INSERM U1111, CNRS UMR5308, ENS de Lyon UCBL Lyon France; ^5^ Laboratoire Cerba, Département d'infectiologie Cerba Healthcare Frépillon France; ^6^ BPO‐BIOEPINE‐Biogroup Levallois‐Perret France; ^7^ Laboratoires Cerballiance Cerba Healthcare Issy‐les‐Moulineaux France; ^8^ National Reference Center for Respiratory Viruses, Institut Pasteur Université Paris Cité Paris France; ^9^ Département de Microbiologie Cerballiance Rhône‐Alpes Lyon France; ^10^ UNILIANS‐BIOGROUP Décines‐Charpieu France; ^11^ Molecular Mechanisms of Multiplication of Pneumovirus, Institut Pasteur, INSERM UMR 1173 (2I), Assistance Publique des Hôpitaux de Paris Université Paris‐Saclay, Université Versailles St Quentin en Yvelines, Université Paris Cité Paris France; ^12^ LABAC Villeurbanne France; ^13^ Plateau Technique Régional Edison Cerballiance Auvergne Rhône‐Alpes Lyon Bron France; ^14^ Mathematical Modelling of Infectious Diseases Unit, Institut Pasteur, U1332 INSERM, UMR2000 CNRS Université Paris Cité Paris France; ^15^ Infectious Diseases Department Santé Publique France Saint‐Maurice France

**Keywords:** community, hospital, influenza, RSV, SARS‐CoV‐2, surveillance network

## Abstract

Influenza, respiratory syncytial virus (RSV) and SARS‐CoV‐2 are among the main respiratory viruses circulating in the population, with a significant burden on public health. While individuals at higher risk are more likely to develop severe symptoms leading to hospitalization, viral circulation in the community remains less extensively monitored. This study compared viral circulation between RELAB, a recently established community‐based laboratory surveillance network (*n* = 22 843 tested patients) and hospital surveillance at the Hospices Civils de Lyon, France (*n* = 23 046 tested patients), for the season 2023–2024. For influenza and SARS‐CoV‐2, similar trends were observed in at‐risk populations (children under 5 years and adults over 65 years) in both settings. Circulation of these two viruses was first detected in the community and insights from the young adult population (19–64 years) were only captured in the community network. Circulation of RSV was more intense in terms of number of cases and started in the pediatric population, and consequently was more represented in the hospital setting. In conclusion, this study highlighted the complementarity between community and hospital surveillance, as different populations and periods of viral circulation were observed.

## 1 Introduction

Influenza, respiratory syncytial virus (RSV), and SARS‐CoV‐2 are among the most significant viral respiratory pathogens globally, contributing substantially to morbidity and mortality, particularly in vulnerable populations such as infants, the elderly, and individuals with preexisting health conditions. Influenza alone is responsible for an estimated 3–5 million cases of severe illness and up to 650 000 respiratory‐related deaths annually worldwide [1]. RSV is a leading cause of acute lower respiratory tract infections, mainly bronchiolitis, in children under 5, accounting for ~3.6 million hospitalizations and 100 000 deaths annually in this age group [2]. In older adults, RSV also contributes significantly to hospitalizations and mortality, with a burden comparable influenza [3]. Since its emergence in late 2019, SARS‐CoV‐2 has caused unprecedented health and societal disruptions, with an estimated 14.9 million excess deaths in 2020 and 2021 alone [4].

Beyond their direct impact on healthcare systems through hospitalizations and medical interventions, these viruses also impose significant indirect costs, including lost productivity, economic disruptions, and strain on public health infrastructures [5]. A critical factor in understanding their public health impact is their distinct seasonality and transmission dynamics. In temperate regions, influenza typically follows a well‐defined winter seasonality, peaking during colder months [6]. RSV also exhibits strong seasonal patterns, often preceding influenza outbreaks and peaking in late autumn or early winter [7]. In contrast, SARS‐CoV‐2 has displayed more variable transmission dynamics, influenced by emerging variants, public health measures, and evolving population immunity [8]. The co‐circulation of these viruses within a single season can result in overlapping waves of infection, compounding pressure on healthcare systems. Furthermore, environmental, behavioral, and biological factors contribute to these distinct patterns, affecting the timing, duration, and intensity of the outbreaks [9].

France's surveillance system for respiratory viral infections, coordinated by Santé Publique France, monitors the circulation and impact of major respiratory viruses, including SARS‐CoV‐2, influenza, and RSV through an integrated, multisource approach. It collects data from general practitioners (réseau Sentinelles), emergency departments (Oscour), home medical emergency services (SOS Médecins), laboratories, intensive care units, elderly care facilities, and wastewater analysis. Weekly reports are published from October to March, combining clinical, virological, and environmental data. The system supports early detection of epidemics, guides public health actions, and informs both professionals and the general public. While the system includes both community‐level and hospital‐based data, it often overlooks the broader burden of disease circulating in the community. Mild and asymptomatic infections, which can serve as reservoirs for transmission, play a critical role in seeding epidemics [10]. Studies comparing hospital‐based and community‐based surveillance have revealed discrepancies in viral circulation, strain prevalence, and patients' demographics, particularly across different age groups and settings [11, 12, 13].

The COVID‐19 pandemic has further disrupted respiratory virus patterns globally. Public health interventions such as mask mandates, social distancing, school closures, and travel restrictions not only suppressed SARS‐CoV‐2 but also significantly altered the seasonality and epidemiology of other respiratory viruses, including RSV and influenza [14, 15]. Following the relaxation of these measures, respiratory viruses have exhibited atypical seasonal dynamics, including off‐season outbreaks and shifts in age‐related vulnerability [16]. However, systematic postpandemic data comparing community and hospital settings for these three viruses remain scarce. Understanding these seasonality dynamics is critical for informing public health responses, vaccine strategies, and healthcare resource planning.

This study leverages data from the RELAB community surveillance network and hospital‐based surveillance at the Hospices Civils de Lyon (HCL) during the 2023–2024 respiratory viral season. The objectives are to compare the circulation of influenza, RSV, and SARS‐CoV‐2 across community and hospital settings, stratified by age. By addressing this critical gap, we aimed to assess the RELAB system's capacity for community‐level monitoring and to demonstrate the complementary value of integrating hospital and community data to provide a comprehensive understanding of viral epidemiology and inform future public health strategies.

## 2 Methods

### 2.1 Study Population

RELAB is a nationwide network aggregating data from two large private community‐based laboratory networks in France, BIOGROUP and CERBALLIANCE, comprising over 1600 sites. In the RELAB network, samples are collected from primary care centers. Established in September 2023, RELAB is coordinated by two national reference centers for viral respiratory infections (NRC‐VIR) at the HCL in Lyon and Institut Pasteur in Paris. For the present study, only laboratories located in the Rhône Department (*n* = 82) were included, covering a population of 1.9 million inhabitants in 2021 [17]. Participating laboratories conducted routine multiplex reverse transcription polymerase chain reaction (RT‐PCR) testing for influenza, RSV, and SARS‐CoV‐2. Virological and demographic information from all tests were electronically transmitted to the NRC‐VIR. The RT‐PCR assays used included, CERBALLIANCE: PKamp Respiratory SARS‐CoV‐2 RT‐PCR Panel Assay (PerkinElmer) (triplex influenza/SARS‐CoV‐2/RSV); BIOGROUP: ID SARS‐CoV‐2/Influenza (duplex influenza/SARS‐CoV‐2) from Week 35 to Week 48, 2023, and EurobioPlex Flucosyn (triplex influenza/SARS‐CoV‐2/RSV) from Week 48, 2023 to Week 13, 2024.

For hospitalized patients at the HCL (the main hospital in the Rhône Department), virological testing was performed using the Panther Fusion SARS‐CoV‐2/Flu A/B/RSV Kit. All samples were collected between August 28, 2023 (Week 35) and March 31, 2024 (Week 13).

### 2.2 Analysis

Statistical analyses were performed using R statistical software (Version 4.4.1). Data were analyzed separately for community or hospital settings.

The number of cases, positivity rate, and total number of tests performed were calculated for each virus, stratified by age group and sex. Numbers of cases were compared between community and hospital data using the *χ* ^2^ test, with a significance level of 0.05. Age pyramids were plotted for each virus with 5‐year age‐groups up to 100 years, with additional categories for children (< 1 year and 1–4 years).

Calculation of the effective reproduction number (Re) over time was performed with the EpiEstim R package [18, 19]. Parametric gamma distributions were used to model the serial intervals, with mean 2.6 and standard deviation 1.5 for influenza [18, 20], mean 7.5 and standard deviation 2.1 for RSV [21], and mean 7 and standard deviation 5.2 for SARS‐CoV‐2 [22]. Daily incidences were aggregated to account for closures on Saturday afternoons and Sundays of the community laboratories. For each virus and setting, numbers of cases were summed on a 7 day‐window each Sunday. Smoothed daily incidences were reconstructed using the EpiEstim R package. For influenza and RSV, reproduction numbers were estimated from the start of the pre‐epidemic period to the end of the post‐epidemic period, as defined by Santé Publique France for the Auvergne‐Rhône‐Alpes region, which is the region of data collection [23]. Santé Publique France does not define epidemic phases at the department level, which is the scale of this study. For influenza, this period lasted from Week 47, 2023 to Week 11, 2024, and for RSV, pre‐epidemic of bronchiolitis started Week 43, 2023 and the epidemic stopped on Week 3, 2024. For SARS‐CoV‐2, as the epidemic was already ongoing when the observation began, the reproduction number was estimated for the whole period. National holidays with school closures happened on Weeks 43 and 44, 2023 (autumn school holidays) and Week 52, 2023 and Week 1, 2024 (Christmas holidays).

## 3 Results

### 3.1 Study Population and Testing

Between Week 35, 2023 and Week 13, 2024, a total of 23 046 patients were tested at the hospital and 22 843 patients were tested in community laboratories for influenza, RSV, and SARS‐CoV‐2. Additionally, 20 337 patients were tested only for influenza and SARS‐CoV‐2 in community laboratories from Week 35 to Week 48 (Table 1). Positivity rates by week were similar for CERBALLIANCE and BIOGROUP (Figure S2). Among positive samples for at least one virus, the majority originated from the community for influenza (78%; *N* = 3912 in community, *N* = 1090 in hospital) and SARS‐CoV‐2 (77%; *N* = 11 353 in community, *N* = 3362 in hospital). For these two viruses, the overall positivity rates in community compared with hospital testing were 1.9 higher for influenza (positivity rates of 9.1% in community and 4.7% in hospital; *p* < 0.001) and 1.7 higher for SARS‐CoV‐2 (positivity rates of 26% in community and 15% in hospital; *p* < 0.001). For RSV, the numbers of positive samples (*N* = 943 in community and *N* = 927 in hospital) and the positivity rates (4.1% in community, 4% in hospital; *p* = 0.6) were similar in both settings. In the community, women accounted for 59% of tested patients, and the positivity rates for each virus were also higher in women (56% of patients positive to influenza, 63% of patients positive to RSV and 61% of patients positive to SARS‐CoV‐2 were women), whereas gender distribution was balanced among hospitalized patients.

**Table 1:** Characteristics of all patients tested and the patients positive either to influenza, RSV, or SARS‐CoV‐2.

	Influenza			RSV			SARS‐CoV‐2		
	Community^a^	Hospital^a^	*p*	Community^a^	Hospital^a^	*p*	Community^a^	Hospital^a^	*p*
Total patients	43 180	23 046		22 843	23 046		43 180	23 046	
Uninterpretable tests	21	14		25	2		21	15	
Positive tests	3912 (9.1%)	1090 (4.7%)	< 0.001	943 (4.1%)	927 (4.0%)	0.6	11 353 (26%)	3362 (15%)	< 0.001
Sex									
M	1709 (44%)	516 (47%)	0.029	351 (37%)	449 (48%)	< 0.001	4392 (39%)	1614 (48%)	< 0.001
Missing	2	2		1	0		7	0	
Age group			< 0.001			< 0.001			< 0.001
0–1	11 (0.3%)	23 (2.1%)		13 (1.4%)	342 (37%)		28 (0.2%)	141 (4.2%)	
1–5	205 (5.2%)	197 (18%)		79 (8.4%)	218 (24%)		60 (0.5%)	78 (2.3%)	
6–18	414 (11%)	99 (9.1%)		75 (8.0%)	38 (4.1%)		499 (4.4%)	58 (1.7%)	
19–64	2550 (65%)	361 (33%)		452 (48%)	117 (13%)		7385 (65%)	953 (28%)	
65+	732 (19%)	410 (38%)		324 (34%)	212 (23%)		3381 (30%)	2129 (63%)	

^a^ *n* or *n* (%) where applicable.

### 3.2 Age Distribution

**Figure 1:**
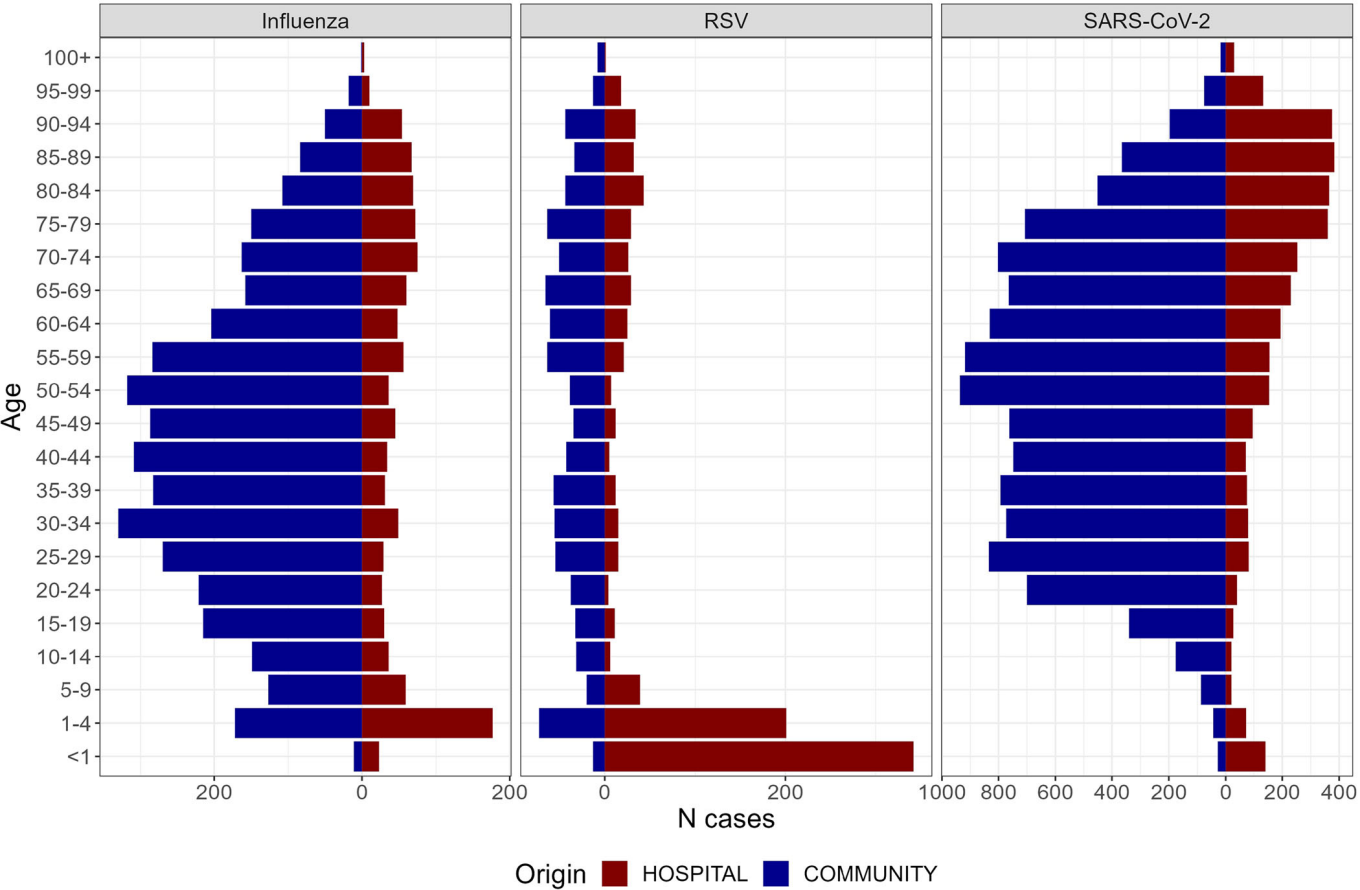
Age pyramid of the patients positive to each virus.

The age distribution of patients testing positive to each virus differed between community and hospital settings (Table 1 and Figure 1). In the community, most positive patients for influenza and SARS‐CoV‐2 were adults aged 19–64 years (65% for both viruses), while for RSV, this age group represented 48% of cases. In contrast, among hospitalized patients, this age group had the second highest rate of positive samples for influenza (33%) and SARS‐CoV‐2 (28%) and was third for RSV (13%). Older adults (≥ 65 years) accounted for the largest proportion of influenza and SARS‐CoV‐2 cases in the hospital, with this age group being twice more represented than in community (38% vs. 19% for influenza, 63% vs. 30% for SARS‐CoV‐2). For RSV, the positivity rate in children under 5 years was 6 times higher in the hospital setting (61%) than in the community (9.8%). Notably, only 13 RSV cases were detected in infants under 1 year in the community. However, BIOGROUP only began RSV testing from Week 48, 2023 so these results may reflect partially the onset of circulation in community. A sensitivity analysis restricted to tests conducted after this time showed similar results (Table S1). Among children under 5 years, the number of influenza‐positive patients was similar in the community (*N* = 216) and the hospital (*N* = 220). Compared to the community, more SARS‐CoV‐2 cases were detected among hospitalized infants under 1 year (*N* = 141, positivity rate of 4.2%; *N* = 28 and positivity rate of 0.2% in community) and children aged 1–4 years (*N* = 78, positivity rate of 2.3%; *N* = 60 and positivity rate of 0.5% in community).

### 3.3 Influenza Circulation

**Figure 2:**
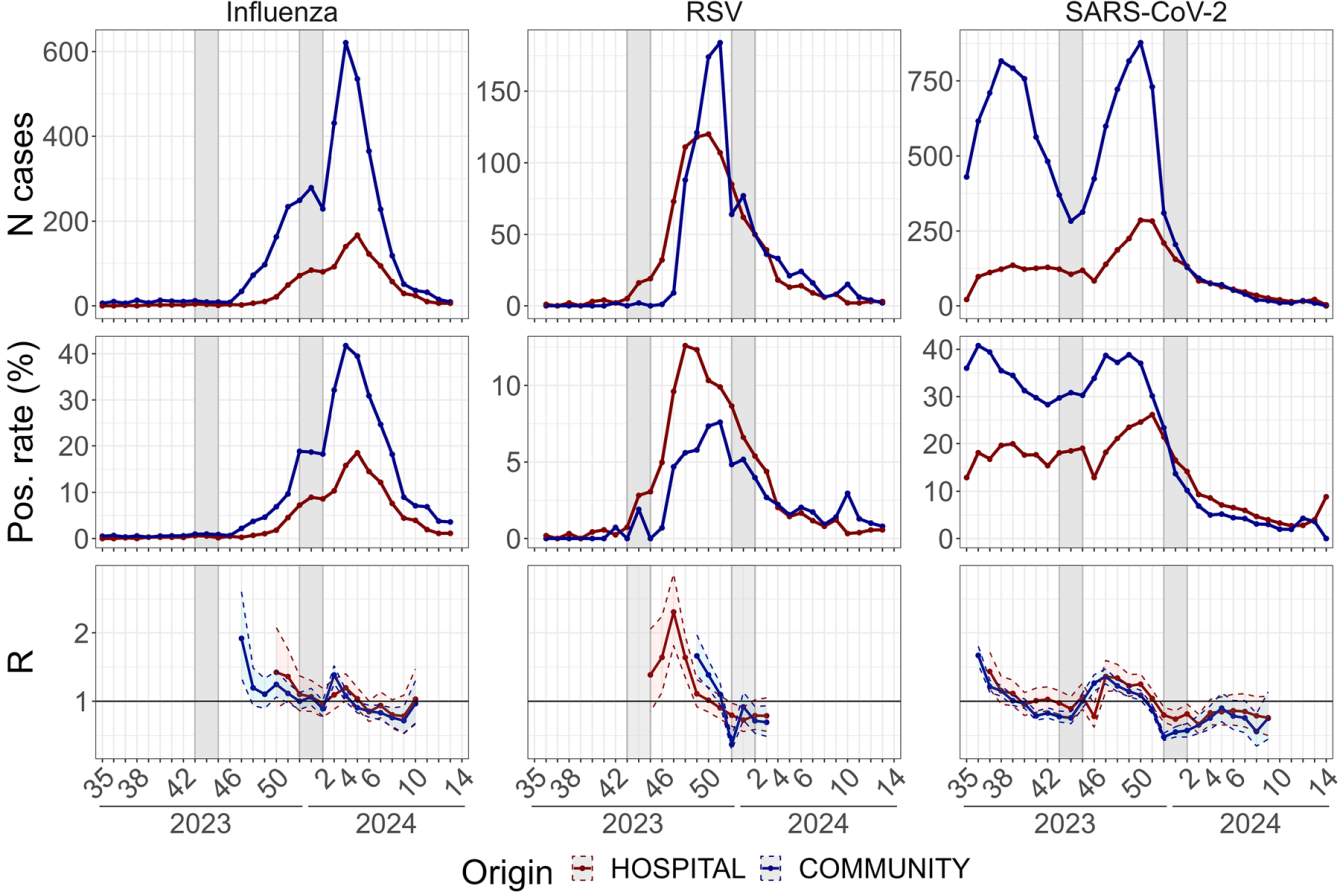
Weekly number of detected cases, positivity rates, and effective reproduction numbers for each virus over time. Pos. rate, positivity rate.

**Figure 3:**
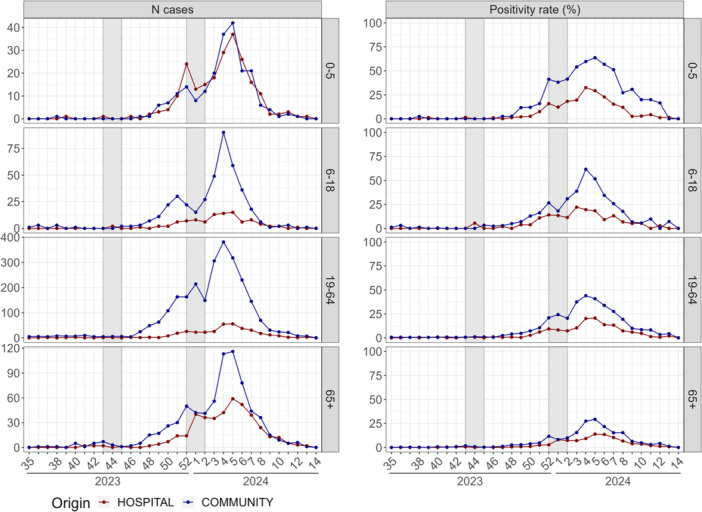
Weekly number of detected cases and positivity rates of influenza by age group over time.

In the community, influenza circulation began in Week 47, 2023, coinciding with the start of the pre‐epidemic period declared in the region by Santé Publique France [23]. During this week, 34 cases were detected in the department, marking a 4.25‐fold increase compared with the previous week (8 cases on Week 46 with a positivity rate of 0.6%). The overall positivity rate reached 2.2% and exceeded 2% across all ages, except in patients over 65 years old (Figures 2 and 3). In the hospital, influenza activity was detected 3 weeks later, on Week 50, with 21 cases and a positivity rate of 1.8%. At this stage, influenza was still in an ascending phase, with positivity rates exceeding 2.5% across all age groups, except for those older than 65 years. Positivity rates at the onset of circulation should, however, be interpreted with caution, as they are based on a few cases and are therefore highly variable. In both settings, similar epidemic trends were observed, with an increase in cases and positivity rates until the Christmas holidays, followed by a plateau and a subsequent postholiday increase. The epidemic peaked in Week 4, 2024 in community and Week 5, 2024 in the hospital, with both settings displaying similar dynamics of epidemic decline and comparable reproduction numbers.

Throughout the season, reproduction numbers aligned between community and hospital settings, indicating synchronized epidemic growth and decline, except from the 1‐week delay in the hospital epidemic peak. However, confidence intervals for the reproduction numbers in the hospital were large due to the limited sample size. Positivity rates were consistently higher in the community than in the hospital, overall and across all age groups. When analyzing age‐specific trends, influenza circulation started in the adult population (19–64 years old) in both settings, followed by adults (≥ 65 years), though with a delay between hospital and community (Figure 3). Influenza circulation was then detected among those aged 6–18 years in the community but not in the hospital. Throughout the season, in both settings, positivity rates by age group followed a decreasing trend with increasing age, with the highest positivity rates observed in the younger patients. However, positivity rates by age should be interpreted with caution, as they were based on a low number of cases, especially in the hospital.

### 3.4 RSV Circulation

**Figure 4:**
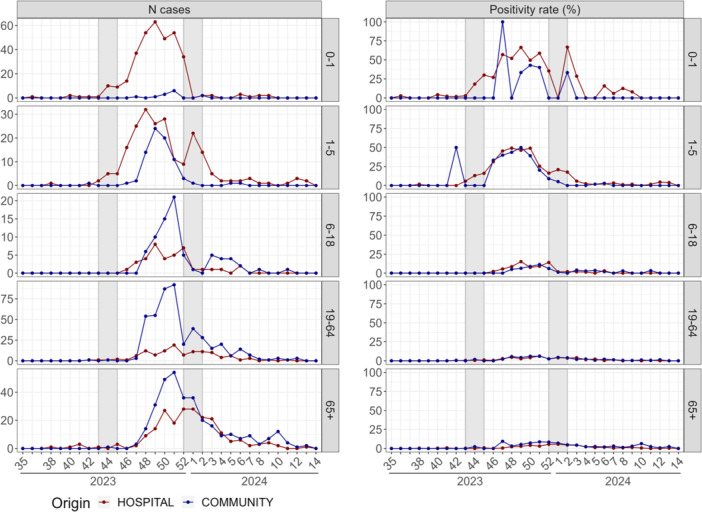
Weekly number of detected cases and positivity rates for RSV by age group over time.

RSV cases were first detected in the hospital on Week 43, 2023, coinciding with the pre‐epidemic phase notification by Santé Publique France [23] (Figure 2). At this stage, the positivity rate among children under 5 years reached 4.3%, and RSV circulation was confirmed the following week, with 15 pediatric cases and a positivity rate of 16.1% (Figure 4). In the community, RSV circulation was detected on Week 47, after the autumn school holidays, with 9 cases and a positivity rate of 4.7% (Figure 2). This observation was based only on CERBALLIANCE data, as BIOGROUP only started systematic RSV testing in Week 48. Throughout the 2023–2024 season, the tested populations differed between setting, with infants in their first year of life being tested almost exclusively in the hospital. RSV circulation was first detected in the pediatric population, and the peak of RSV detection was delayed only by 1 week in the community (Week 51) compared to the hospital (Week 50) (Table 1 and Figures 1 and 4). The decrease in circulation of RSV began prior to the Christmas holidays, and the number of cases observed during the final phase of the epidemic remained relatively similar in both settings.

In the hospital, the reproduction number could be evaluated early, and when estimated simultaneously with community data, it differed between settings. During the season, positivity rates were consistently higher in the hospital compared with the community overall and among children under 5 years (Figures 2 and 4). In contrast, for adults over 65 years, positivity rates were higher in the community than in the hospital, and detection started 1 week earlier in the community (Figure 4). Very few cases of young adults (19–64 years old) were detected in the hospital compared with the community, and detection among children aged 6–18 years was low in both setting (Table 1 and Figures 1 and 4).

### 3.5 SARS‐CoV‐2 Circulation

**Figure 5:**
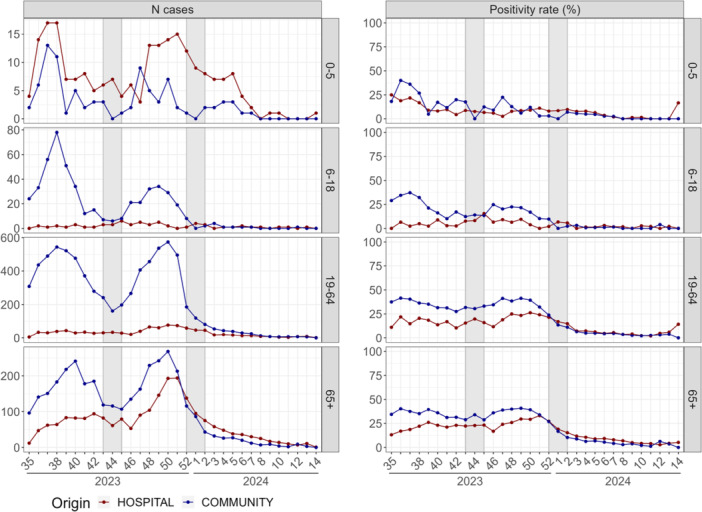
Weekly number of detected cases and positivity rates infected by SARS‐CoV‐2 by age group over time.

Circulation of SARS‐CoV‐2 was already ongoing at the start of the observation period in Week 35, 2023, with 430 cases detected and a positivity rate of 36% in the community and 21 cases and a positivity rate of 13% in the hospital (Figure 2). The number of cases increased over the following 4 weeks, leading to two distinct waves of SARS‐CoV‐2 circulation in the community. The first wave was followed by a rebound that started in the community during the autumn school holidays and 2 weeks later in the hospital. In the first wave, only a slight increase in hospital cases was observed for patients over 65 years (Figure 5). The second wave peaked in Week 50, 2023 simultaneously in the community and the hospital. The decline in case numbers and positivity rates after the second peak was very similar in both settings (Figure 2). Additionally, the reproduction numbers for the second wave were comparable, reflecting similar transmission dynamics in both settings.

SARS‐CoV‐2 detection patterns were similar in both settings for patients over 65 years, especially regarding the dynamics of circulation in the second wave (Figure 5), even if positivity rates were higher in the community until the peak of the second wave. As for the other viruses, young adults (19–64 years old) accounted for the highest number of cases in the community. Positivity rates of both groups of adults (19–64 and ≥ 65 years) were similar in the community but not in the hospital. SARS‐CoV‐2 circulated in the community among patients 6–64 years, while this age group was less represented in the hospital, likely reflecting a lower hospitalization rate in this population.

### 3.6 Viral Circulation During the National Holidays

The detections of influenza and RSV were less impacted by Christmas holidays in the hospital than in community. Although influenza circulation was still in an ascending phase, a decrease in the number of community cases, with a reproduction number lower than 1, was observed on Week 2, 2024, the second week of the Christmas holidays. However, since this drop coincided with a reduction in testing volume in the community (Figure S1) while positivity rates remained stable (Figure 2), it remains unclear whether the decline in cases was driven by a true epidemic decline or a change in testing habits. This reduction in the number of cases was not observed in hospital surveillance.

For RSV, a slight reduction in the number of cases and positivity rate in the community was noted during the first week of the Christmas holidays, likely due to fewer tests being conducted (Figure 2 and Figure S1). In contrast, in the hospital, the number of cases, positivity rate, and reproduction number remained unaffected by the holiday period, suggesting that hospital‐based detection patterns were not influenced by holidays‐related decreases in testing.

The reduction in the number of tests for SARS‐CoV‐2 during the national holidays did not seem to impact the detection in the community. However, the autumn school holidays occurred between the two epidemic waves and the decrease of the second wave was already advanced during the Christmas holidays.

## 4 Discussion

This study provided a comprehensive analysis of respiratory virus dynamics during the 2023–2024 season, comparing community‐based (RELAB) and hospital‐based (HCL) surveillance data within a single French department. The findings highlighted the complementary value of these two data sources in understanding the spread and impact of influenza, RSV, and SARS‐CoV‐2.

The results from the first season of the RELAB network highlighted the added value of this community laboratories network compared with hospital surveillance. In particular, similar trends with the hospital data were observed for the below 5 years and over 65 years groups for influenza and SARS‐CoV‐2, both in terms of number of cases and reproduction numbers. For all viruses, community data offered insights that were not captured by hospital data in the young adult population (19–64 years old). Specifically, for influenza and SARS‐CoV‐2, community‐based surveillance captured earlier and broader circulation in young adults, an age group less likely to be hospitalized. This finding aligns with previous studies showing that community‐based surveillance often detects viral circulation earlier than hospital systems [11]. Hospital data, on the other hand, provided essential insights into vulnerable populations, especially older adults and children under 5 years, who have higher hospitalization rates. These trends are consistent with Shi and colleagues, who emphasized the disproportionate burden of respiratory viruses among these age groups [3]. SARS‐CoV‐2 circulation was already ongoing when the study started and, surprisingly, the first peak of SARS‐CoV‐2 cases was not observed in the hospital setting. A previous study on the circulation of SARS‐CoV‐2 in France found that community and hospital surveillance provided similar information, but with a lag of ~6 days [22].

Hospital monitoring of RSV provided more information compared with community surveillance on the number of cases and positivity rates in the population under 5 years, particularly in infants in their first year of life. This may be critical for early detection of RSV epidemics, which typically start in this age group [24]. In particular, HCL is the only hospital with a pediatric intensive care unit in the Lyon urban area and therefore admits the most severe cases of RSV infection. For RSV, detection began earlier in the hospital, with the majority of cases occurring in those under 1 year. This underscores the importance of hospital‐based surveillance for tracking severe pediatric cases.

The timing of the first detected cases in both community and hospital was consistent with the regional pre‐epidemic periods as defined by Santé Publique France [23]. For influenza and RSV, epidemic curves and reproduction numbers demonstrated how the two data sets complement each other. The impact of holidays on testing behaviors was evident in the community data, where a reduction in community testing was observed during holiday periods. This reduction led to challenges in interpreting trends during these periods. Conversely, hospital numbers remained stable, providing more consistent insights. This observation aligns with previous studies which noted the resilience of hospital‐based data during periods of reduced community engagement in testing and the stability of reproduction numbers when estimated on severe cases [25].

A notable limitation of this study was that it focused on a single epidemic season in one geographical area. This limits the generalizability of the findings, as viral dynamics can vary across regions and seasons. Including data from additional seasons and hospitals as well as expanding the RELAB network to other geographic areas would strengthen these conclusions. Zachariah and colleagues emphasized the need for longitudinal studies to validate early findings in community surveillance [11]. At this local level, reproduction numbers were estimated from small numbers of cases. Another limitation was the delayed implementation of RSV testing in one laboratory network, which restricted the ability to fully capture community RSV dynamics. In addition, differences in viral strains and vaccination rate between hospital and community were not assessed in the present study.

Despite these limitations, the findings have significant implications for public health and virological surveillance. The complementary nature of community and hospital data highlights the importance of integrating community‐based and hospital‐based surveillance systems. Community‐based surveillance effectively detected first influenza and SARS‐CoV‐2 circulation, offering critical early warnings for public health interventions. Meanwhile, hospital surveillance provided valuable insights into severe cases, particularly among at‐risk patients. This dual approach offers a more holistic understanding of respiratory virus epidemics, as noted in prior studies [16] which emphasized the need for layered surveillance systems in post‐COVID‐19 respiratory virus monitoring [26].

Looking forward, expanding the RELAB network and collecting data across multiple seasons will enhance its representativeness and utility for public health. Such efforts will allow for better identification of epidemic onsets criteria and better understanding of viral transmission across age groups. Additionally, as new viral variants emerge, continuous monitoring of their impact on hospitalization rates and community transmission is crucial for adapting public health strategies. This is consistent with previous recommendations to incorporate variant‐specific data into ongoing surveillance efforts [12].

In conclusion, community and hospital surveillance systems provided complementary information on the dynamics of respiratory viruses. Community‐based surveillance allowed for early detection of influenza and SARS‐CoV‐2 circulation, while hospital data provided more detailed insights into severe cases, especially for RSV in the pediatric population. Future research should aim to validate these findings across broader geographic areas and multiple seasons, ensuring these data sources can effectively inform public health responses and resource allocation during respiratory virus epidemics.

## Ethics Statement

The study protocol was approved by the Institutional Review Board of the Hospices Civils de Lyon (Comité Scientifique et Éthique des Hospices Civils de Lyon) on June 2023 (Reference 23‐5039). Unique anonymized alphanumeric identifiers were used. Data transfer to the research team was performed through a secured portal, accessible only to the research team.

## Consent

According to the French regulation, informed consent was not necessary for noninterventional studies. However, all patients or parents of minors received an information notice detailing the nature and use of the collected data. If a patient or parent declined the data to be used, all corresponding data were removed from the research database.

## Conflicts of Interest

M.C.N. reports grants from Sanofi and personal fees from Pfizer and Sanofi. The other authors declare no conflicts of interest.

## Supporting information


**Supporting Table 1:** Characteristics of all patients tested and the patients positive to RSV from Week 48 of 2023. **Supporting Figure 1:** Weekly numbers of tests by virus in the community and in the HCL by age group. **Supporting Figure 2:** Weekly number of positivity rates by virus in the community according to the laboratory of the RELAB network.

## Data Availability

Part of the data supporting the findings of this study can be requested from the corresponding author, subject to approval by the RELAB study group.
