# Supplementary material for: Community and Hospital‐Based Laboratory Surveillance for Influenza, Respiratory Syncytial Virus, and SARS‐CoV‐2 During the 2023–2024 Season, Lyon, France
Source: J Med Virol. 2025 Aug 29;97(9):e70549. doi: 10.1002/jmv.70549 (PMC12396161; doi:10.1002/jmv.70549)

**Supporting information for the manuscript entitled “Community and hospital-based laboratory surveillance for influenza, RSV, and SARS-CoV-2 during the 2023-2024 season, Lyon, France”**

Supplementary Table 1: Characteristics of all patients tested and the patients positive to RSV from week 48 of 2023.

|  |  | **RSV** | | |  |
| --- | --- | --- | --- | --- | --- |
|  |  | **COMMUNITY***^1^* | **HOSPITAL***^1^* | **p** |  |
| **Total patients** | | 21,655 | 14,718 |  |  |
| Uninterpretable tests | | 24 | 0 |  |  |
| **Positive tests** | | 925 (4.3%) | 749 (5.1%) | <0.001 |  |
|  | **Sex** |  |  |  |  |
|  | M | 346 (37%) | 356 (48%) | <0.001 |  |
|  | Missing | 1 | 0 |  |  |
|  | **Age group** |  |  | <0.001 |  |
|  | 0-1 | 12 (1.3%) | 256 (34%) |  |  |
|  | 1-5 | 74 (8.0%) | 157 (21%) |  |  |
|  | 6-18 | 75 (8.1%) | 34 (4.5%) |  |  |
|  | 19-64 | 444 (48%) | 103 (14%) |  |  |
|  | 65+ | 320 (35%) | 199 (27%) |  |  |

*^1^*n or n (%) where applicable

Supplementary Figure 1: Weekly numbers of tests by virus in community and in the HCL by age group


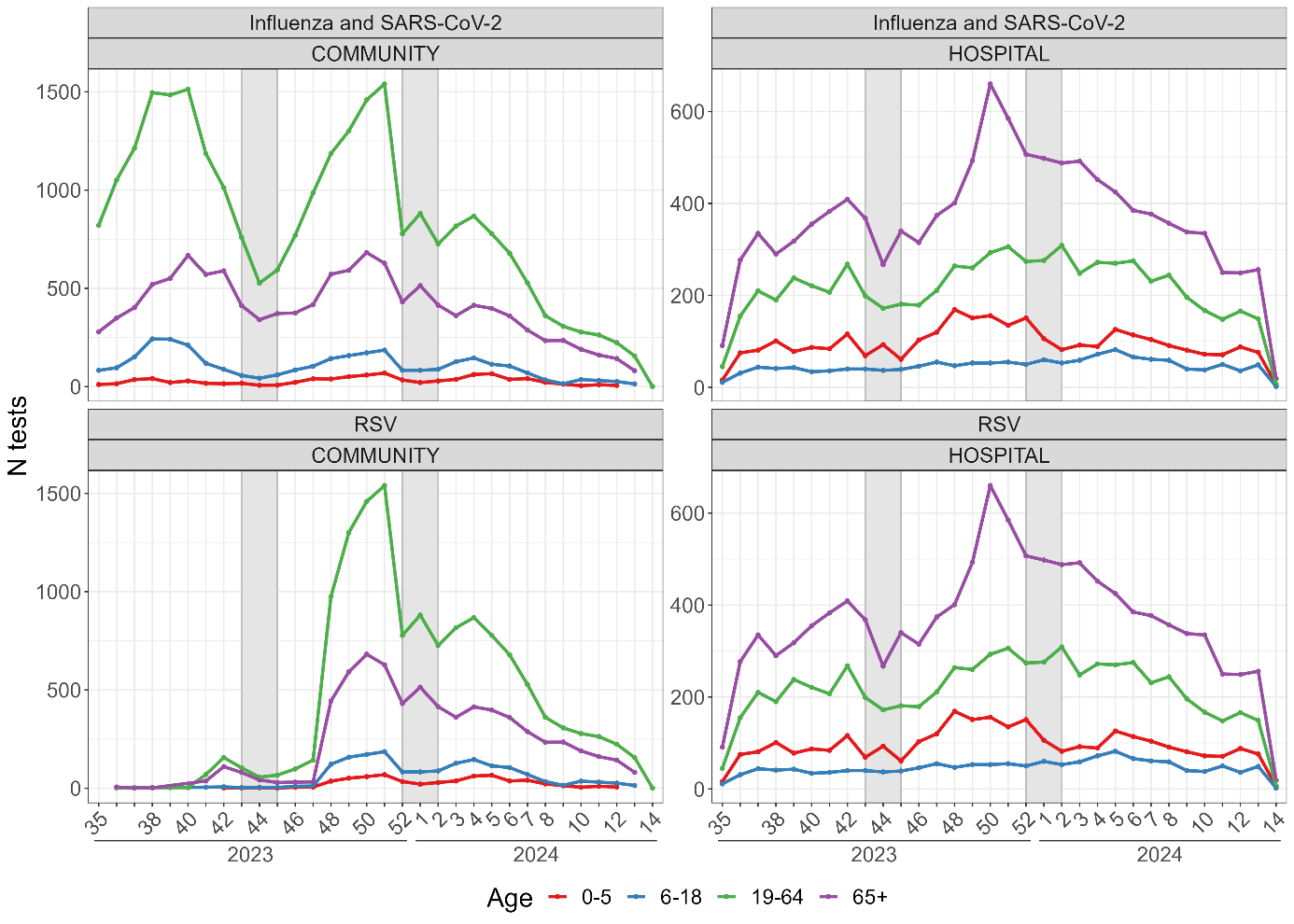


Supplementary Figure 2: Weekly number of positivity rates by virus in the community according to the laboratory of the RELAB network


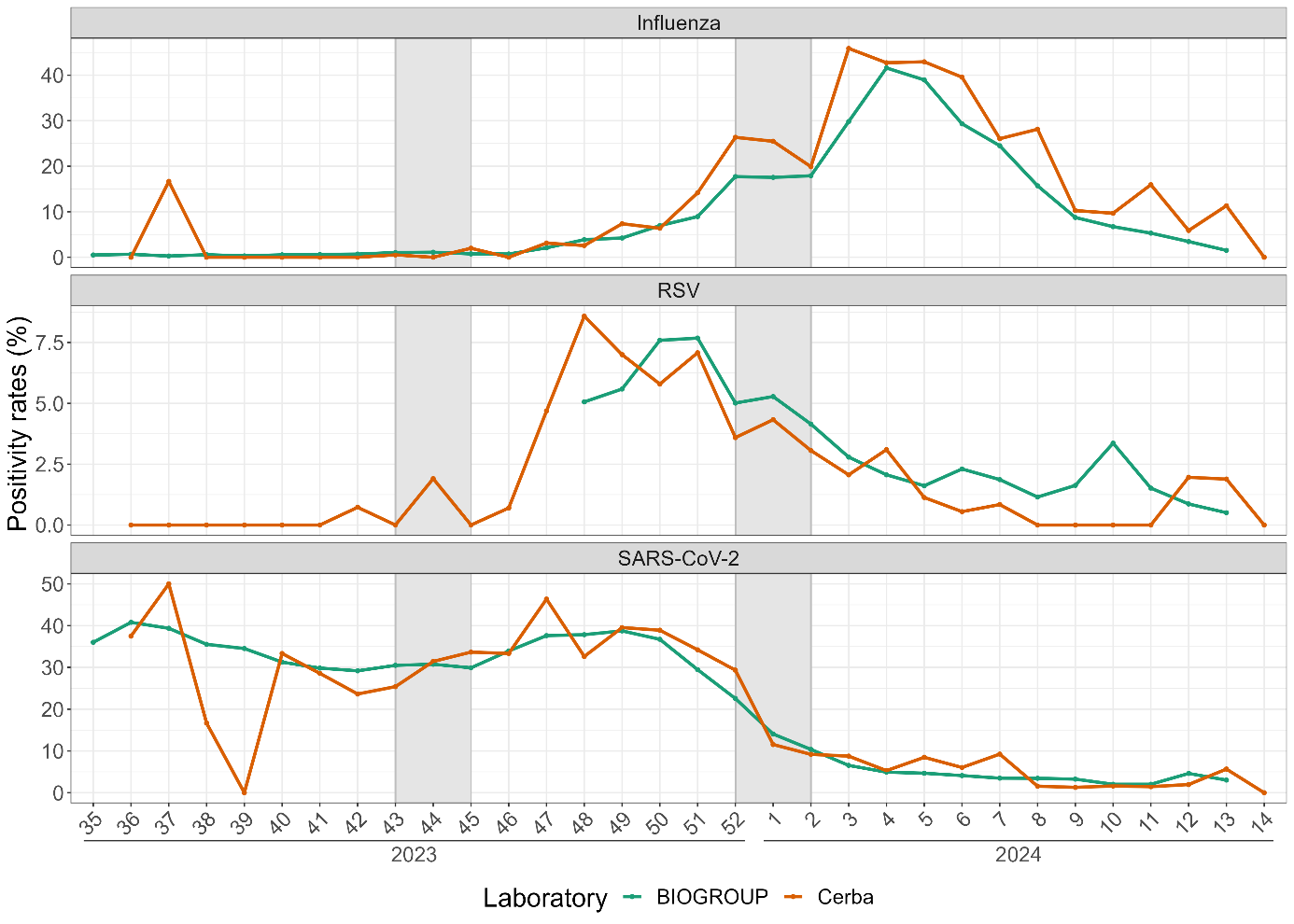

Supplement: Supplementary file 1 — Supporting Table 1: Characteristics of all patients tested and the patients positive to RSV from Week 48 of 2023. Supporting Figure 1: Weekly numbers of tests by virus in the community and in the HCL by age group. Supporting Figure 2: Weekly number of positivity rates by virus in the community according to the laboratory of the RELAB network. [file JMV-97-e70549-s001.docx]
